# Identification of R2R3-MYB Gene Family and Functional Analysis of Responses of S22 Subfamily to Abiotic Stresses in Dandelion (*Taraxacum mongolicum* Hand.-Mazz.)

**DOI:** 10.3390/ijms26073422

**Published:** 2025-04-05

**Authors:** Liangruinan Lu, Songle Fan, Bi Qin, Jingang Wang, Lifeng Wang, Shizhong Liu

**Affiliations:** 1 College of Horticulture and Landscape Architecture, Northeast Agricultural University, Harbin 150006, China; luliangruinan@163.com; 2 Key Laboratory of Biology and Genetic Resources of Rubber Tree, Ministry of Agriculture and Rural Affairs, Rubber Research Institute, Chinese Academy of Tropical Agricultural Sciences, Haikou 571101, China; slfan@catas.cn (S.F.); qinbi126@163.com (B.Q.); lfwang@catas.cn (L.W.)

**Keywords:** R2R3-MYB, *Taraxacum mongolicum* Hand.-Mazz., abiotic, saline alkaline, transcription factor

## Abstract

Dandelions possess a wide range of medicinal properties and demonstrate remarkable adaptability and tolerance to salinity and alkalinity. *MYB* genes in plants are implicated in growth, differentiation, metabolism, and responses to both biotic and abiotic stresses. The function of MYB genes in dandelions, particularly the R2R3-MYB gene family, requires further investigation. In this study, we identified a total of 130 members of the dandelion R2R3-MYB gene family at the genome-wide level, all of which were mapped to eight dandelion chromosomes. MEME analysis revealed that TmR2R3-MYB proteins contain three conserved motifs. Phylogenetic analysis categorized all TmR2R3-MYBs into 29 subfamilies. Transcriptomic studies in different tissues indicated that TmR2R3-MYBs exhibit distinct expression patterns in different tissues, indicating their diverse functions in dandelions. Notably, *TmMYB44* from the S22 subfamily displayed the highest expression level in roots. Additionally, six representative TmR2R3-MYBs were selected from the S22 subfamily for expression profiling under salinity and alkalinity treatments. The results demonstrated that the TmR2R3-MYBs from the S22 subfamily are involved in the response to salinity and alkalinity stress. These findings provide a basis for further exploration of the functions of TmR2R3-MYBs in abiotic stress tolerance.

## 1. Introduction

Dandelions (*Taraxacum mongolicum* Hand.-Mazz.) are perennial herbs belonging to the Asteraceae family [1]. Dandelions are found worldwide and exhibit a high degree of adaptability, allowing them to thrive in harsh environments [2]. The growth and development of dandelions are influenced by a variety of biotic and abiotic stresses, including salinity, wind, chill, and drought. This species acts as a Zn excluder [3] and a cadmium accumulator [4]. Notably, dandelions are recognized for their salt tolerance, making them well suited for extensive cultivation [5] in Northeast China and Korea [2] due to their effective components [6,7,8], which include polysaccharides, flavonoid glycosides, flavonoid derivatives, phenylpropanoids, benzoic acid derivatives, and polymethoxylated flavones. These characteristics position dandelions as promising candidates for applications such as nitric oxide inhibition [9] and hepatoprotection [10].

The V-myb avian myeloblastosis viral oncogene homolog (MYB) transcription factor (TF) superfamily is the largest protein superfamily. It is characterized by its versatile functions and presence in all eukaryotes [11]. Unlike fungi and animals, plants possess a significantly higher number of MYB genes. Members of the MYB protein family share a common feature: they contain a highly conserved DNA-binding domain composed of 1–4 incomplete repeats (Rs), each forming a helix–turn–helix (HTH) motif that includes three regularly spaced tryptophan residues, totaling approximately 50–55 amino acid residues. Based on the number of conserved MYB domains, members of this protein family are classified as 1R-MYB (consisting of one or two separated repeats), 2R-MYB (R2R3-MYB, consisting of two adjacent repeats), 3R-MYB (consisting of three adjacent repeats), or 4R-MYB (consisting of four adjacent repeats) [12].

The MYB TF family plays a crucial regulatory role in plant growth and development, as well as in responses to biotic and abiotic stresses. Dandelions exhibit a unique tolerance to saline–alkaline conditions, making it significant to study the members of the R2R3-MYB family involved in the transcriptional regulation of abiotic stress. R2R3-MYB members have been extensively investigated due to their roles in the transcriptional regulation of abiotic stress in both model and non-model plants. For example, the R2R3-MYB MYB8 regulates the response to iron-deficiency stress in *Arabidopsis thaliana* [13]. Compared to the wild type (Col-0), overexpression of the *MYB37* (more than 100-fold) OE-1 and OE-2 lines significantly alleviates symptoms of salt stress in *Arabidopsis* [14]. Under high-NaCl conditions, the *Arabidopsis myb3* mutant, which was completely undetectable by RT-PCR analysis, had longer root growth than wild-type plants [15]. MYB73 is involved in regulating the organization of actin filaments in response to salt stress in *Arabidopsis.* Its expression level was upregulated by 8-fold 12 h after treatment with 100 mM NaCl [16]. In comparison to the wild type (WT), overexpression of *IbMYB330* by about 7-fold in roots enhances drought and salt stress tolerance in transgenic tobacco [17]. Expression of the poplar gene *PagMYB151* increases by 60- to 120-fold under salt stress treatments, facilitating proline accumulation and thereby enhancing salt tolerance [18]. Overexpression of the grape gene *VhMYB2* increases tolerance to salinity and drought [19]. Additionally, overexpression of the *CgMYB1* gene from *Chenopodium glaucum* in *Arabidopsis* enhances both salt and cold tolerance [20]. Furthermore, the subgroup 22 (S22) member of the MYB TF family, *MYB73,* plays key roles in phosphate deficiency tolerance [21], salt stress tolerance [22], and resistance to fungal pathogens [23]. Investigating the specific functions of MYB TFs in dandelions, particularly the roles of S22 members in salinity stress, will be of great significance.

In this study, using bioinformatics tools, a total of 130 members belonging to the TmR2R3-MYB subfamily in dandelions were identified. This study encompassed an examination of the physicochemical properties, conserved motifs, three-dimensional structure, and chromosomal localization of the TmR2R3-MYB gene family. Furthermore, the classification and expression profiles of the TmR2R3-MYB S22 subfamily were analyzed across various tissues and under different saline–alkaline stresses, laying the groundwork for future investigations into the functional roles of TmR2R3-MYB genes in dandelions.

## 2. Results

### 2.1. Identification, Physicochemical Properties, and Chromosomal Mapping of 130 TmR2R3-MYB TFs in Dandelions

**Figure 1:**
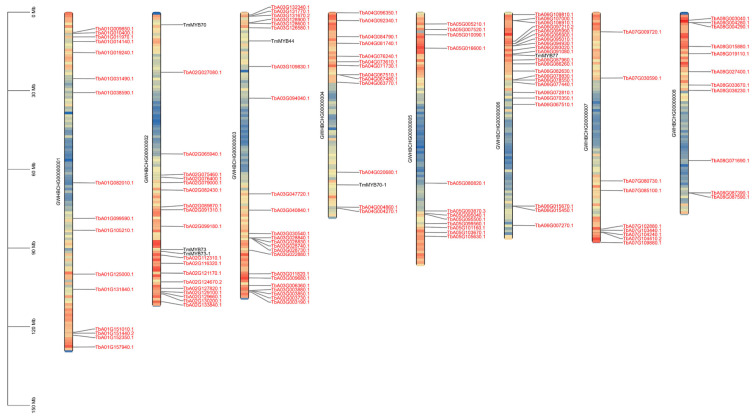
The distribution of 130 *TmR2R3-MYB* genes across the chromosomes of dandelions. The colored modules on the chromosomes indicate the density of the genes. MYB genes labeled in black represent members of the S22 subfamily, while those labeled in red represent other *TmR2R3-MYB* genes.

As shown in Figure 1, a total of 130 unique R2R3-MYB family genes (Supplementary Table S1) were identified in the dandelion genome. The *TmR2R3-MYB* genes were predominantly mapped to chromosome 6 (Chr06), followed by Chr02 and Chr03. In order to further understand the molecular characteristics of the Tm R2R3-MYB genes, a physicochemical property analysis was conducted. The lengths of the amino acids encoded by Tm R2R3-MYB ranged from 114 to 1140, the isoelectric points varied from 4.84 to 10.18, and the theoretical molecular weights ranged from 13.55 to 124.76 kDa.

### 2.2. Gene Structure and Sequence Analysis of TmR2R3-MYBs

**Figure 2:**
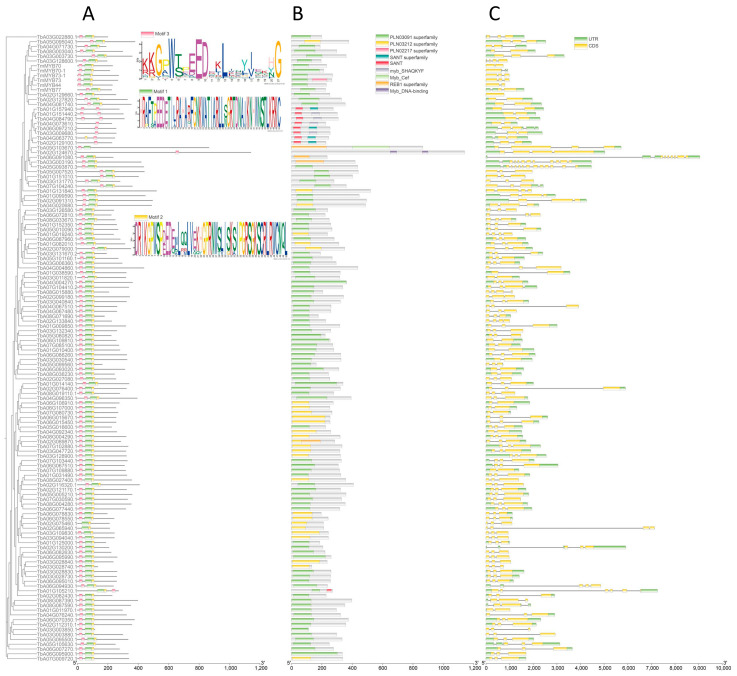
Analysis of the (**A**) motifs, (**B**) conserved domains, and (**C**) gene structure of the 130 TmR2R3-MYB sequences.

To investigate the characterizations of the homology domains of TmR2R3-MYB proteins, as well as the frequencies of amino acids at each position within each repeat of the MYB domain, we utilized the online MEME tool to search for conserved motifs shared among these proteins and constructed a phylogenetic tree. To clearly illustrate the order of the TmR2R3-MYB proteins and group markers in each motif plot, we arranged them according to their sequence in the phylogenetic tree (Figure 2A). In this study, we retrieved the largest motif common to 130 dandelion protein sequences for the first time. The arrangement order of the motifs for the 130 TmR2R3-MYBs is 3, 1, 2 (Figure 2A). The conserved domains of the 130 TmR2R3-MYBs are shown in the figure, indicating that some MYB domains have undergone variations but still belong to the MYB family (Figure 2B). In terms of gene structure, the number of exons ranges from 2 to 12 (Figure 2C).

### 2.3. Phylogenetic Analysis of TmR2R3-MYBs

**Figure 3:**
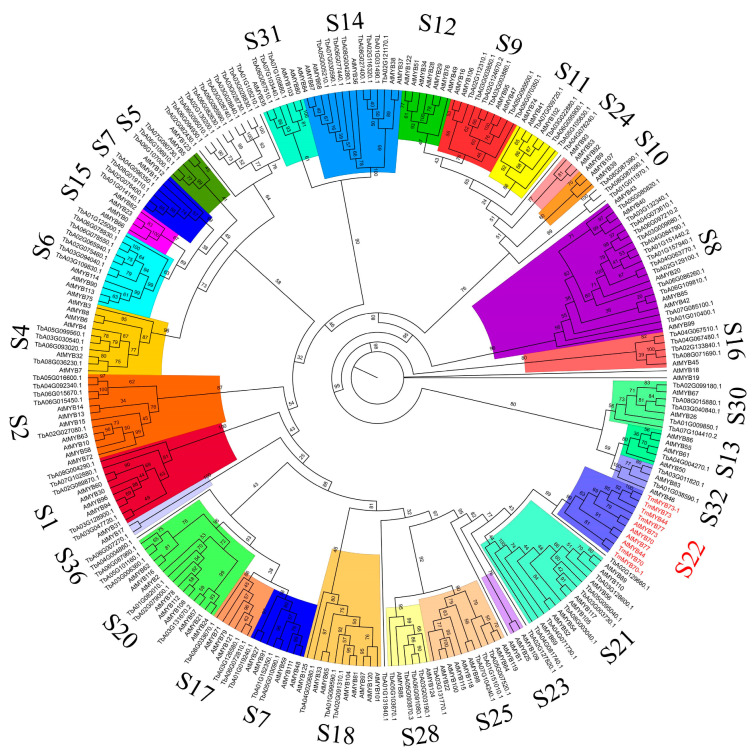
Phylogenetic tree of 130 TmR2R3-MYBs with 126 AtR2R3-MYBs generated by Maximum Likelihood method. Branches with different colors indicate different subfamilies. S22 subfamily marked with red color.

In order to infer the evolutionary relationships among the dandelion R2R3-MYB families, the 130 Tm R2R3-MYBs were clustered with 126 members of the Arabidopsis AtMYB family from *Arabidopsis thaliana* (Supplementary Table S2 and Figure 3). Based on these results and previous studies of the *Arabidopsis thaliana* AtMYB families, the 130 TmMYBs were classified into 29 subfamilies [24].

### 2.4. Expression Profiles of TmR2R3-MYBs in Different Tissues

**Figure 4:**
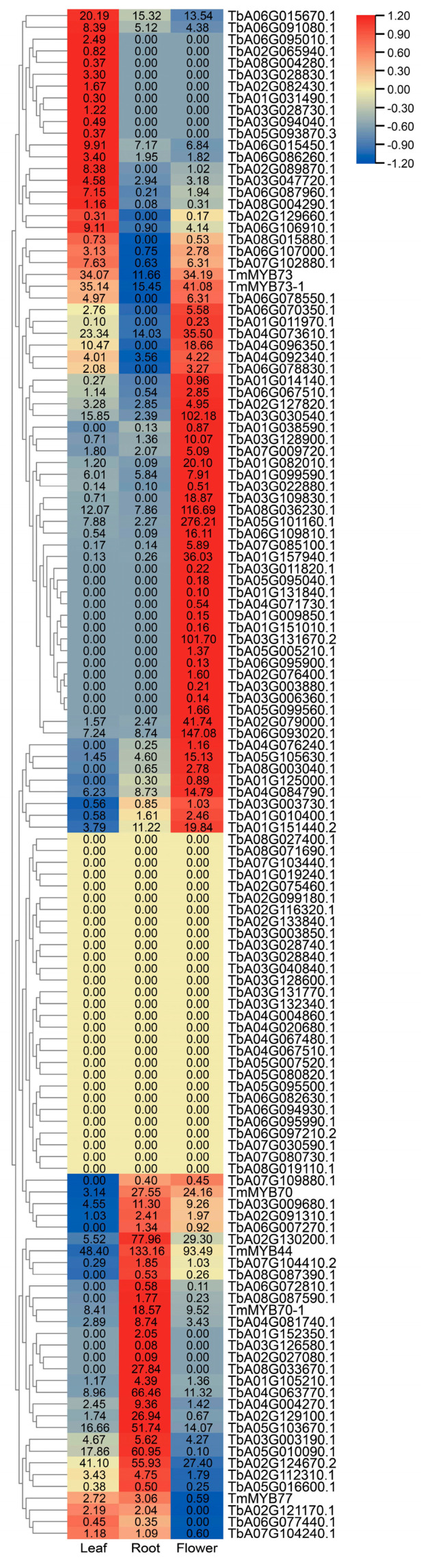
The expression levels of the 130 TmR2R3-MYB genes in leaves, roots, and flowers are presented. The values in the heatmap squares are expressed as FPKM, normalized using the row scale, and are represented in different colors.

The expression levels of TmR2R3-MYBs in different tissues of dandelions, including leaves, roots, and flowers, are shown in Figure 4 and Supplementary Table S3. The genes that were highly expressed in the leaves were mainly *TmMYB44*, *TbA02G124670.2*, *TmMYB73-1*, and *TmMYB73*. Most of them were from the S22 group. The genes that were highly expressed in the roots were mainly *TmMYB44* (S22), *TbA02G130200.1*, and *TbA04G063770.1* (S8). The genes that were highly expressed in the flowers were mainly *TbA05G101160.1* (S20), *TbA06G093020.1* (S4), and *TbA08G036230.1* (S4). Members of the S22 subfamily, *TmMYB44*, *TmMYB70*, *TmMYB470-1*, and *TmMYB77*, were all highly expressed in the roots. Notably, the expression level of TmMYB44 in the dandelion roots was the highest among the S22 subfamily.

### 2.5. Expression Profiles of S22 Subfamily Members After Subjecting Dandelions to Different Saline–Alkaline Stress Treatments

**Figure 5:**
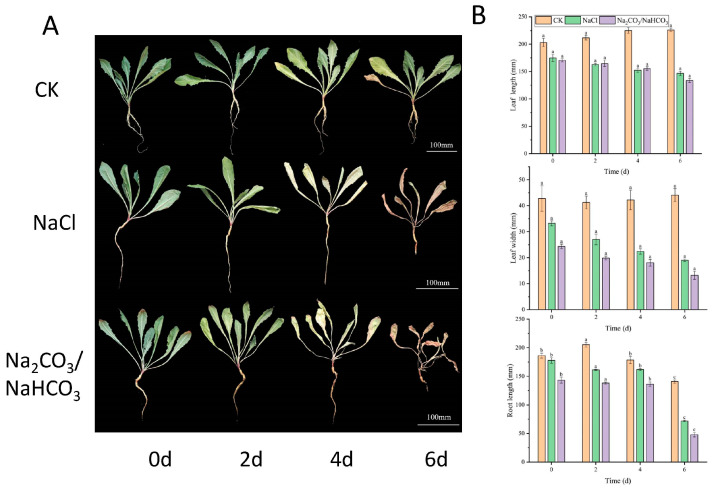
(**A**) The phenotypes and (**B**) the leaf lengths, leaf widths, and root lengths of dandelions treated with 0.3 mol·L^−1^ NaCl and a saline–alkaline solution with a pH of 10.78. Different lowercase letters indicate significant differences at the *p* < 0.05 level.

Different saline–alkaline stress treatments were applied to dandelions, including 0.3 mol·L^−1^ NaCl and a saline–alkaline solution with a pH of 10.78. Leaf samples were photographed at 0 d, 2 d, 4 d, and 6 d following the saline–alkaline stress treatments (Figure 5A). As shown in Figure 5B, after exposure to salt–alkali stress, the leaf length, leaf width, and root length of the salt–alkali treatment group gradually decreased compared to the control group as the days progressed.

**Figure 6:**
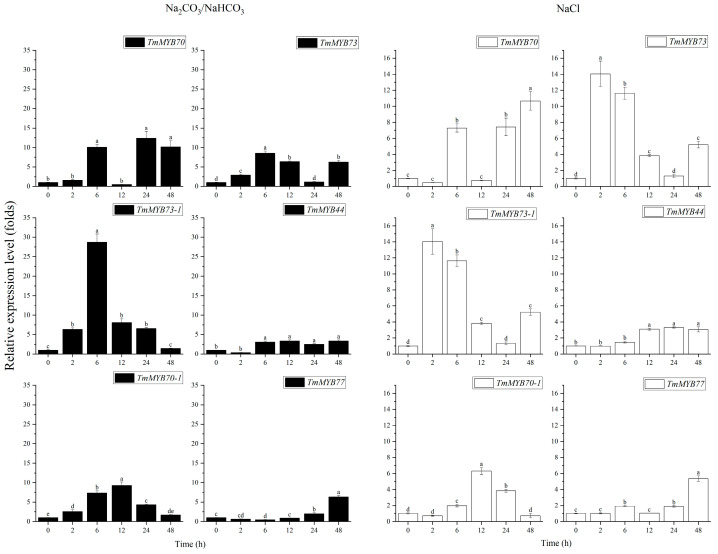
The expression levels of 6 TmMYBs from the S22 subgroup under Na_2_CO_3_/NaHCO_3_ and NaCl treatments. Different lowercase letters indicate significant differences at the *p* < 0.05 level.

As illustrated in Figure 6, treatment with a mixed solution of Na2CO_3_/NaHCO_3_ at pH 10.78 significantly increased the expression levels of six TmMYBs from the S22 subfamily in dandelions. Specifically, the expression levels of *TmMYB73*, *TmMYB73-1*, and *TmMYB70-1* exhibited initial increases, followed by decreases over time, peaking at 6 h and 12 h. In contrast, the expression levels of *TmMYB70*, *TmMYB44*, and *TmMYB77* consistently increased over time, reaching their maximum levels at 24 h and 48 h. Following treatment with a 0.3 mol·L^−1^ NaCl solution, the expression levels of the six TmMYBs from the S22 subfamily observed in dandelions were also significantly elevated. The expression patterns of *TmMYB73*, *TmMYB73-1*, and *TmMYB70-1* again showed increases followed by decreases, peaking at 6 h and 12 h. Meanwhile, the expression levels of *TmMYB70*, *TmMYB44*, and *TmMYB77* continued to rise, reaching their highest levels at 24 h and 48 h. Overall, the expression patterns of the S22 subfamily in dandelions were similar under treatments with both saline and alkaline–saline solutions.

## 3. Discussion

In China, there are approximately 100 million hectares of saline–alkaline land [5]. The potential for dandelions to be utilized as a cost-effective strategy for the reclamation of saline–alkaline land in China is significant [5]. All three types of saline–alkaline stress inhibited the growth of sunflowers to various degrees, with the inhibitory effects ranked in the following order: NaCl combined with Na_2_SO_4_ exhibited the greatest inhibition, followed by NaCl combined with NaHCO_3_ and finally NaCl alone [25]. The findings regarding the impact of salt–alkali stress on rice growth indicate that there are notable differences between neutral stress (NaCl) and alkaline stress (Na_2_CO_3_ and NaHCO_3_). The observed order of effect severity is as follows: Na_2_CO_3_ > NaHCO_3_ > NaCl [26]. Our results also indicated that neutral stress (NaCl) had a more obvious effect than alkaline stress (Na_2_CO_3_ and NaHCO_3_) regarding growth inhibition in dandelions (Figure 5).

The MYB family is a group of transcription factors that are widely distributed across all eukaryotes [27]. To date, numerous MYB genes have been identified in various plant species, and MYB TFs have been shown to play crucial roles in plant development, secondary metabolism, hormone signaling, disease resistance, and abiotic stress tolerance [28,29,30]. MYB TFs have been found to play critical roles in plant responses to salt stress related to their physical properties, gene structures, and sequence differences. For instance, the salt tolerance-related protein STO binds to an MYB transcription factor homologue, conferring salt tolerance in *Arabidopsis* [31]. Subsequently, an increasing number of MYB TFs and their underlying mechanisms, which are activated in response to salt stress, have been elucidated. TaMYB33 enhances the ability to balance osmotic pressure and scavenge reactive oxygen species (ROS), thereby increasing salt tolerance [32]. Additionally, *AtMYB74* is regulated by the RNA-directed DNA methylation pathway in response to salt stress [33]. An RNA gel blot analysis indicated that the expression of the *AmMYB1* transcript was significantly elevated in green photosynthetic tissues. Furthermore, this expression was markedly enhanced in response to various stressors, including salt (500 mM), light (500 µE m^−2^ s^−1^), and the external application of abscisic acid (ABA) at a concentration of 100 µM [34]. Under control (CK) conditions, the growth of yeasts harboring LpMYB4 was comparable to that of the control group. However, when subjected to stress treatments involving 1 M NaCl, 30 mM NaHCO_3_, 20 mM Na2CO_3_, and 3.4 mM H_2_O_2_, the growth of yeast strains with overexpressed *LpMYB4* demonstrated a significant improvement relative to the control [35].

In this study, a total of 130 dandelion Tm R2R3-MYB genes were identified, and the expression of 6 TmMYBs in the S22 subfamily was analyzed (Figure 1 and Figure 6). TmMYBs of the S22 subfamily were ubiquitously expressed in the cells of all examined dandelion organs (Figure 4). Among them, *TmMYB44* exhibited the highest expression level in the roots, suggesting that these genes may have distinct roles in different plant tissues (Figure 4). Wang et al. systematically reviewed the role of MYB44 in plants, elucidating its responses to both biotic and abiotic stresses, as well as its regulatory network, which influences plant metabolism and development [36]. Following salt treatment, the expression level of *RmMYB44* in the roots and leaves of *Rosa multiflora* initially increased and then decreased over time. Overexpression of *RmMYB44* in tobacco plants enhanced their resistance to cold, drought, and salt stress [37]. Salt stress induces the eviction of H2A.Z-containing nucleosomes from the AtMYB44 promoter region, which may weaken its affinity for the AtMYB44 protein, a repressor of *AtMYB44* gene transcription under conditions without salt stress [38]. *MYB44* gene expression significantly decreases under salinity-stress conditions in sunflowers [39]. In conclusion, this study suggests that TmMYB44 plays a crucial role in the saline–alkaline resistance of dandelions.

The S22 subgroup of R2R3-MYBs in plants exhibits a unique ability to tolerate salt. Our findings demonstrated that the expression of six TmMYBs from the S22 subgroup was influenced by Na2CO_3_/NaHCO_3_ and NaCl treatments (Figure 6). RT-PCR profiling revealed that the expression of *TaMYB73* in the roots of wheat significantly increased between 0.5 h and 24 h following exposure to 200 mM NaCl. In contrast, the transcript levels in the leaves were lower than those observed in the non-stressed control samples [40]. In *Arabidopsis*, the S22 subgroup comprises four members: MYB44, MYB73, MYB70, and MYB77. MYB77 plays a role in modulating auxin signal transduction [41] and is regulated by the ABA receptors PYL9 and PYL8 [42,43]. In this study, we identified six TmMYBs from the S22 subgroup, highlighting the significance of the S22 subgroup in dandelions. MYBs play a direct role in salt tolerance, as evidenced in other plant species. GhMYB73 was first identified through transcriptomic analysis of cotton subjected to a salt treatment. The observed upregulation of GhMYB73 following exposure to salt stress suggests that this gene may be integral to the plant’s response to abiotic stressors [44]. GhMYB73 physically interacts with both GhPYL8 and AtPYL8, suggesting that GhMYB73 regulates ABA signaling during the response to salinity stress [22].

In conclusion, a total of 130 TmMYB family members were identified in dandelions. Expression analysis revealed that most TmMYB members of the S22 subfamily were predominantly expressed in floral tissues, while a few were expressed in root and leaf tissues. Notably, the expression level of TmMYB44 was highest in the roots, and the expression of the TmMYBs from the S22 subgroup was significantly upregulated following a saline–alkaline stress treatment. TmMYB44 may play an important role in the saline–alkaline resistance of dandelions.

## 4. Materials and Methods

### 4.1. Plant Materials and Treatments

Dandelion cultivars were preserved at the Rubber Research Institute of the Chinese Academy of Tropical Agricultural Sciences, Haikou City, Hainan Province, China (19°59′0″ N; 110°19′20″ E). The experimental material consisted of dandelion strain Tb tissue culture seedlings, which were planted in the laboratory’s artificial climate chamber. The incubation temperature was maintained at 23 °C, with a light period of 16 h per day and a light intensity ranging from 100 to 200 μmol m^−2^ s^−1^. After two months of growth, hydroponic fixation was conducted for 3 days, during which the roots of plants exhibiting consistent growth were immersed in a solution of 0.3 mol·L^−1^ NaCl and a saline–alkaline solution with a pH of 10.78. The specific treatment methods were based on protocols established in our laboratory [45]. Plant samples were photographed 0, 2, 4, and 6 days after the different saline–alkaline stress treatments. Leaves were collected 0, 2, 6, 12, 24, and 48 h post-treatment, and the samples were rapidly frozen in liquid nitrogen to analyze the expression patterns of 6 TmMYB genes in the S22 subfamily under saline–alkaline and salt treatments.

### 4.2. Identification of TmR2R3-MYB Gene Family

Nucleotide and protein sequences from dandelions were downloaded from the Genome Warehouse (GWH) database https://ngdc.cncb.ac.cn/gwh/Assembly/19733/show (accessed on 6 June 2024). The HMMER (PF00249) and Blastp (E-value: 10^−5^) were used to identify members of the MYB family in the dandelion genome [46]. False-positive genes were removed using the conserved domains tool available on the NCBI website. The structure domains of MYBs were predicted using the online tool SMART [47] http://smart.embl-heidelberg.de/smart/set_mode.cgi?NORMAL=1 (accessed on 6 June 2024), where MYBs containing two SANT domains were classified as members of the R2R3-MYB family.

### 4.3. Analysis of TmR2R3-MYBs’ Physicochemical Properties and Conserved Sequence

A conserved domain analysis of MYB proteins was conducted using the NCBI conserved domain http://www.ncbi.nlm.nih.gov/Structure/cdd/wrpsb.cgi? (accessed on 6 June 2024) and SMART http://smart.embl-heidelberg.de/smart/set_mode.cgi?NORMAL=1 (accessed on 6 June 2024) to identify the structural characteristics of TmMYBs. The isoelectric point, molecular weight, instability index, and hydrophilicity of the identified genes were analyzed by uploading the amino acid sequences of the TmMYB members to the ExPASy ProtParam tool https://web.expasy.org/protparam/ (accessed on 6 June 2024) [48]. Sequence alignment was performed using DNAMAN version 8.0 and ClustalX [49]. The conserved motifs shared by MYB proteins were analyzed using the Multiple Em for Motif Elicitation MEME v5.0.5, http://meme-suite.org/tools/meme (accessed on 6 June 2024) [50] online tool by uploading the amino acid sequences of TmMYB family members.

### 4.4. Phylogenetic Analysis of TmMYBs

A phylogenetic tree analysis used 126 Arabidopsis R2R3-MYB protein sequences (Supplementary Table S2) from the TAIR database. Muscle [51] was used to align the amino acid sequences of 126 AtMYB protein sequences with 130 TmR2R3-MYB protein sequences. A phylogenetic tree was constructed using the MYB subfamily proteins from *Arabidopsis thaliana* and dandelions. Phylogenetic analysis was conducted using IQ-TREE software v2.4.0 [52], employing the Maximum Likelihood method, with a bootstrap test performed using 1000 replicates [53].

### 4.5. Analysis of Dandelion Tissue-Specific Expression Patterns

The genome assembly sequence and annotation file of dandelions (GWHBCHG00000000) were downloaded from the Genome Warehouse (GWH) database. The dandelion transcriptome data (SRR29313597, SRR29313596, SRR29313595, SRR29313591, SRR29313590, SRR29313589, SRR29313594, SRR29313593, and SRR29313592) of different tissues and the genome were downloaded from the NCBI [1]. FastQC [54] and Trimmomatic [55] were used to analyze the sequencing quality and to filter the raw data to obtain clean reads. The clean reads were aligned to the corresponding dandelion reference genome using HISAT2 software v2 2.2.1 [56]. Subsequently, the transcriptome sequence data of dandelion tissues, including leaves, roots, and flowers, were assembled and quantified using StringTie software v3.0.0 [57]. Information on reads aligned to the dandelion reference genome was used to count the number of reads aligned to the genome using the featureCounts tool in the subread software v2.0.7, providing read count values for dandelion genes [58]. FPKM (Fragments Per Kilobase of transcript sequence per Million base pairs sequenced) values were calculated for each *TmMYB* gene in different dandelion tissues based on gene sequence lengths. Differential expression analysis between dandelion leaf, root, and flower tissues was conducted using the DESeq2 tool [59]. Gene expression patterns were shown by heatmaps.

### 4.6. RNA Extraction and Analysis of Gene Expression Pattern

A Total RNA Isolation System Kit (OMEGA) was used to isolate total RNA from different tissues. First-strand cDNA was synthesized from 2 μg of total RNA using a RevertAid™ First-Strand cDNA Synthesis Kit (Fermentas Inc, Burlington, ON, Cadana). Quantitative real-time PCR (RT-qPCR) was conducted on a CFX96™ Real-Time System (Bio-Rad, Hercules, CA, USA) with a total reaction volume of 25 μL, employing SYBR Premix Ex Taq (TaKaRa, Tokyo, Japan). Six pairs of specific primers (see Table S3) were designed for the reaction, with GAPDH serving as the internal reference gene. Each treatment included three biological replicates and three technical replicates per sample, and the expression levels were analyzed using the 2^−ΔΔCT^ method [55]. All data were subjected to a one-way ANOVA, and Tukey’s test was applied for multiple comparison analysis at the *p* < 0.01 level. The primers are listed in Supplementary Table S4.

### 4.7. Statistical Analysis

All obtained data were statistically analyzed using the SPSS software package (version 27.0). A one-way ANOVA, along with Tukey’s test, was employed to assess statistical significance at the *p* < 0.05 probability level. The means of three replicates and their corresponding standard deviations (SDs) are reported.

## Data Availability

This manuscript includes the essential data either as figures or as Supplementary Materials.

## Supplementary Materials

The following supporting information can be obtained at https://www.mdpi.com/article/10.3390/ijms26073422/s1.
